# Evaluation of Antidiabetic Properties of the Leaves Extract of *Ficus vallis-choudae* Delile in a Model of Type 2 Diabetes Induced by High-Fat Diet and Streptozotocin

**DOI:** 10.1155/2021/1502230

**Published:** 2021-12-08

**Authors:** Kilenma Kolefer, David Miaffo, Roger Ponka

**Affiliations:** ^1^Department of Biological Sciences, Faculty of Sciences, University of Maroua, Cameroon. P.O. Box 814, Maroua, Cameroon; ^2^Department of Life and Earth Sciences, Higher Teachers' Training College, University of Maroua, P.O. Box 55, Maroua, Cameroon; ^3^Department of Agriculture, Livestock and Derivated Products, National Advanced School of Engineering of Maroua, University of Maroua, P.O. Box 46, Maroua, Cameroon

## Abstract

This work aimed to determine the phytochemical composition of the aqueous extract of leaves of *Ficus vallis-choudae* (AEFV) and to evaluate its antidiabetic properties on a model of type 2 diabetes induced by a high-fat diet (HFD) and a low dose of streptozotocin (STZ). The phytochemical analysis was carried out according to several methods using the standard of each bioactive compound. Type 2 diabetes was induced by feeding rats for 4 weeks with HFD lard followed by injection of a low dose of STZ (35 mg/kg). After induction, the rats were divided into groups and treated for 28 days with metformin (40 mg/kg) and the AEFV at doses of 110, 220, and 440 mg/kg. The results showed that the AEFV contains saponins, flavonoids, tannins, and total polyphenols. In addition, it dramatically reduced body mass, body mass index (BMI), atherogenic index (AI), coronary heart risk index (CRI), and abdominal fat and increased homeostatic model assessment of *β*-cell function (HOMA-*β*), high-density lipoprotein cholesterol (HDL-c) levels, and cardioprotective index (CI). The AEFV also lowered blood glucose levels, insulinemia, homeostatic model assessment of insulin resistance (HOMA-IR) index, and total cholesterol (TC), triglycerides (TG), low-density lipoproteins cholesterol (LDL-c), and very-low-density lipoproteins cholesterol (VLDL-c) levels. There was a decrease in alanine aminotransferase (ALT) and aspartate aminotransferase (AST) activity and in urea and serum creatinine levels following the administration of AEFV. The AEFV caused increased superoxide dismutase (SOD) and catalase (CAT) activities, reduced glutathione (GSH) levels, and decreased malondialdehyde (MDA) levels in the liver, kidneys, and heart of rats. The AEFV has hypoglycemic, antioxidant, and cardioprotective properties, thus validating its use in traditional medicine for the treatment of type 2 diabetes and its complications.

## 1. Introduction

Diabetes is a serious chronic disease that occurs when the body cannot produce or does not make enough insulin or cannot use what it does effectively. The main types of diabetes mellitus are type 1 and type 2. Type 2 diabetes is the most common form of diabetes with around 90% of cases worldwide [1]. It is a very heterogeneous disease that begins with a progressive decline in the action of insulin then with an irreversible deterioration in the functioning of the *β*-cells of the pancreas [2, 3]. Insulin resistance is frequently linked to obesity or circulating free fatty acids because it is clearly established that an increase in the number of lipids in peripheral nonadipocyte tissues is responsible for the phenomenon of insulin resistance [4]. According to the International Diabetes Federation report, 463 million people worldwide suffered from diabetes in 2019 and 700 million are expected in 2045. In Africa, 19 million people suffered from diabetes in 2019, and by 2045, the number of diabetics will reach more than 47 million [1].

The goals of treatment for diabetes mellitus are to keep blood sugar around its normal value and thus prevent its metabolic complications. In the case of type 2 diabetes, hygiene and dietary measures constitute the first components of treatment by instituting a balanced diet moderately low in calories, rich in dietary fiber and unsaturated fats [5], as well as regular physical activity. In the event of the ineffectiveness of the hygiene and dietetic measures, the use of oral antidiabetics is considered. This is the case with metformin, whose mechanism of action is based on the inhibition of hepatic production and intestinal absorption of glucose [6]. However, the excessive cost and the side effects of these antidiabetics, as well as the insufficiency of medical infrastructures, associated with the lack of healthcare personnel in Africa are pushing the populations to turn to traditional medicine.

According to the World Health Organization, about 80% of the world population in developing countries, for socioeconomic and cultural reasons, mainly depend on medicinal plants to treat themselves and to treat mild illnesses (colds, stomach aches, etc.) than severe (cancer, malaria, diabetes, etc.) [7]. These plants constitute an inexhaustible resource that provides the majority of the active principles of pharmaceutical products. However, for rational use of these plants, work must be carried out to determine the bioactive components and the possible harmful effects induced by the use of these and which could lead to other complications in the treatment of pathologies [8].

*Ficus vallis-choudae* (*F. vallis-choudae*) Delile is a tropical and subtropical shrub or tree of the Moraceae family found in Cameroon, Senegal, and Ethiopia [9]. A decoction of its leaves and young leafy stems are used as local medicines for jaundice, nausea, bronchial, and gastrointestinal disorders [10]. Its figs are edible and very popular with children [11]. The extract of its bark has antifungal and anticonvulsant activities [12, 13] as well as anti-inflammatory and antinociceptive effects [14]. In Cameroon, the leaves of *F. vallis-choudae* are traditionally used to treat diabetes mellitus. This work aimed to determine the phytochemical composition of the aqueous extract of leaves of *Ficus vallis-choudae* (AEFV) and to evaluate its antidiabetic properties on a model of type 2 diabetes.

## 2. Materials and Methods

### 2.1. Plant Material

The plant material used in this study consisted of the leaves of *F. vallis-choudae* Delile (Moraceae) collected in Koza (Far North, Cameroon). A sample of this plant has been identified and authenticated at the National Herbarium of Yaoundé (Cameroon) and registered under number 5115 SRF/Cam. Then, the collected leaves were dried in the shade and crushed until a fine powder was obtained.

### 2.2. Preparation of the AEFV

After harvesting and drying the leaves of *F. vallis-choudae*, 19.8 g of powder was introduced into 0.5 L of distilled water for maceration of 24 hours. Once macerated, the mixture was filtered using Whatman No. 1 paper. The filtrate was evaporated in an oven at 45°C for 72 hours to obtain the crude mass of the extract (2.50 g), i.e., an extraction yield of 12.62%.

The daily amount of *F. vallis-choudae* that the traditional healer gives to adult patients is 2500 mg. This mass of extract supposedly consumed by an adult of 70 kg allowed us to calculate the human therapeutic dose, which is 35.71 mg/kg. The equivalent dose in rats was approximately 220 mg/kg, calculated according to the formula of Reagan-Shaw et al. [15]. The doses 110, 220, and 440 mg/kg were used for this test. The duration for the chronic diabetes test is usually 21 to 28 days.

### 2.3. Phytochemical Screening

#### 2.3.1. Total Phenol Content

Total phenol content was determined by using the Folin–Ciocalteu reagent [16]. 0.5 mL of AEFV or gallic acid (standard), 2.5 mL of the Folin–Ciocalteu reagent (10%), and 4 mL of sodium carbonate (7.5%, w/v) were mixed and incubated for 30 min at room temperature. The absorbance of the mixture was measured at 727 nm. Total phenol content was expressed as milligrams of gallic acid equivalents per gram (mg GAE/g) of extract.

#### 2.3.2. Total Flavonoid Content

Total flavonoids content was determined according to the method described by [17]. 1 mL of AEFV or quercetin (standard), 0.2 mL of aluminum chloride (10% w/v), 0.2 mL of potassium acetate (1 M), and 5.6 mL distilled water were mixed well and incubated for 30 min at room temperature. The absorbance of the mixture was measured at 415 nm. Total flavonoid content was expressed as milligrams of quercetin equivalents per gram (mg QE/g) of extract.

#### 2.3.3. Total Tannins Content

The determination of total tannin content was carried out according to the protocol of Bainbridge et al. [18]. 50 *μ*L of catechin (standard) or AEFV, 750 *μ*L of chloride acid solution (12 M), and 1.5 mL of methanol (4%) were mixed and incubated for 20 min at room temperature. The absorbance of the mixture was read at 500 nm. The total tannin content was expressed in milligrams of catechin equivalent per gram (mg CE/g) of extract.

#### 2.3.4. Total Saponin Content

The total saponins content was determined using the method described by Makkar et al. [19]. 50 *µ*L of AEFV or diosgenin (standard), 250 *µ*L of distilled water, 250 *µ*L of vanillin reagent (4%), and 2.5 mL of sulfuric acid (72%) were mixed well and kept in a water bath at 60°C for 10 min. After cooling in ice-cold water, the absorbance of the mixture was read at 544 nm. The total saponins content was expressed as milligrams of diosgenin equivalents per gram (mg GAE/g) of extract.

### 2.4. Animal Material

The animal material consisted of male albino rats of the Wistar strain, aged 16 to 17 weeks and weighing between 200 and 250 g. Only male rats are used in this study to avoid the effects of female rat sex hormones, which may interfere with the results. Rats were reared at the animal house of the Department of Biological Sciences of the University of Ngaoundéré (Cameroon). Animals were housed in polypropylene cages under standard environmental conditions (temperature 22 ± 2°C in a light/dark cycle of 12 h) and fed with HFD and water ad libitum. They were acclimatized for 14 days under laboratory conditions before the start of the test. All animal experiments were handled according to the Cameroon National Ethics Committee (Ref. No. FWIRB 00001954), and all experiments were examined and approved.

### 2.5. Induction of Type 2 Diabetes

To induce type 2 diabetes, the animals were divided into 2 groups, a group of rats given a normal diet consisting of 5% fat, 52% carbohydrate, and 20% protein and the second group of animals given a diet rich in fat (58%), carbohydrates (17%), and protein (25%) [20]. After 30 days, only rats with a body mass index (BMI) > 0.7 g/cm^2^ each received an intraperitoneal injection of streptozotocin (STZ) (diluted in 0.01 mol/L of sodium citrate buffer, pH 4.4) at a single dose of 35 mg/kg. Immediately, the rats were administered a glucose solution (5%) orally to prevent glycemic shock. Three days later, rats with blood glucose greater than 126 mg/dL were selected for the study.

### 2.6. Distribution and Treatment of Animals

Thirty (30) rats were divided into 6 groups of 5 rats each and treated for 28 days as follows:

  Group 1 (control normal rats): normal diet + distilled water (10 mL/kg)
  Group 2 (diabetic control rats): HFD + distilled water (10 mL/kg)
  Group 3 (standard control rats): HFD + metformin (40 mg/kg)
  Group 4: HFD + AEFV (110 mg/kg)
  Group 5: HFD + AEFV (220 mg/kg)
  Group 6: HFD + AEFV (440 mg/kg)

The rats were weighed at the start of the experiment and then every week for 28 days using an electric balance.

The BMI was calculated on the first day and at the end of the induction using the formula: BMI = body mass (g)/size^2^ (cm^2^). The size was taken with a ruler from the muzzle to the anus of the rat.

### 2.7. Collection of Blood and Organs

On the last day of treatment, the animals were fasted for 24 hours, anesthetized by an intraperitoneal injection of ketamine (50 mg/kg bw) and diazepam (10 mg/kg bw).The abdominal cavity has been opened, and the blood sample was collected in tubes without anticoagulant and centrifuged at 3000 rpm for 20 min at 4°C. The supernatant obtained was taken and stored at −20°C for the assay of the biochemical parameters. After collecting the blood samples, organs such as the liver, kidneys, and heart were removed and stored for the determination of antioxidant parameters.

### 2.8. Biochemical Analysis

Glycemia was measured using a “one-touch” glucometer at the start of the experiment and then weekly for 28 days. Serum insulin levels were determined using a rat enzyme-linked immunosorbent assay insulin kit. Homeostasis model assessment of insulin resistance (HOMA-IR) was calculated according to the Matthews et al. [21] method using the formula: HOMA-IR = insulin (*μ*g/L) × glycemia (mg/dL)/22.4. Homeostatic model assessment of *β*-cell function (HOMA-*β*) was calculated using the formula: HOMA-*β* = 20 × insulin (*μ*IU/mL)/FBS (mmol/L) − 3.5) [22]. Total cholesterol (TC), triglycerides (TG), low-density lipoproteins cholesterol (LDL-c) and very-low-density lipoproteins cholesterol (VLDL-c), and urea were assayed according to the method of Kaplan [23, 24]. Malondialdehyde (MDA) was assayed according to the method of Yagi [25]. Reduced glutathione (GSH) was determined according to the method of Weckbercker and Cory [26]. Superoxide dismutase (SOD) was assayed by the method of Misra and Fridovish [27]. Catalase (CAT) was assayed according to the method of Aebi [28]. Aspartate aminotransferase (AST), alanine aminotransferase (ALT), and creatinine were determined according to the method of Murray [29–31]. The atherogenic index (AI) was calculated using the formula: AI = Log (TG/HDL-c) [32]. The coronary heart risk index (CRI) was calculated according to the methods of Barter et al. [33] using the formula: CRI = TC/HDL-c. The cardioprotective index (CI) was calculated using the following formula: CI = LDL-c/HDL-c [34].

### 2.9. Statistical Analysis

The results were expressed as mean ± standard derivation. Data were analyzed using one-way analysis of variance (ANOVA) followed by Tukey's post hoc test and two-way ANOVA followed by the Bonferroni post-test using Graph Pad Prism version 5.0 software. A value of *p* < 0.05 was considered statistically significant.

## 3. Results

### 3.1. Phytochemical Study of AEFV

The composition of bioactive components of AEFV is summarized in Table 1. The results revealed the presence of several secondary metabolites such as polyphenols, saponins, tannins, and flavonoids. The contents of AEFV in polyphenols, flavonoids, tannins, and saponins were 67.06 ± 0.11 mg AGE/g, 37.55 ± 0.11 mg QE/g, 31.80 ± 0.09 mg EC/g, and 17.60 ± 0.05 mg DE/g, respectively. However, the content of saponins was relatively lower than the contents of polyphenols, tannins, and flavonoids. In addition, the AEFV has a high concentration of total polyphenols compared with the other components.

### 3.2. Effect of AEFV on Body Weight, BMI, and Abdominal Fat

Table 2 shows the effect of AEFV on body weight, BMI, and abdominal fat. It appears that the body weight, the BMI, and abdominal fat of the animals in the diabetic control group increased significantly (*p* < 0.001), compared with the normal control group. Treatment with AEFV and metformin significantly (*p* < 0.001) decreased the body weight, BMI, and abdominal fat, compared with the untreated diabetic control.

### 3.3. Effect of AEFV on Blood Glucose, Insulinemia, and HOMA-IR and HOMA-ß Indices

**Figure 1:**
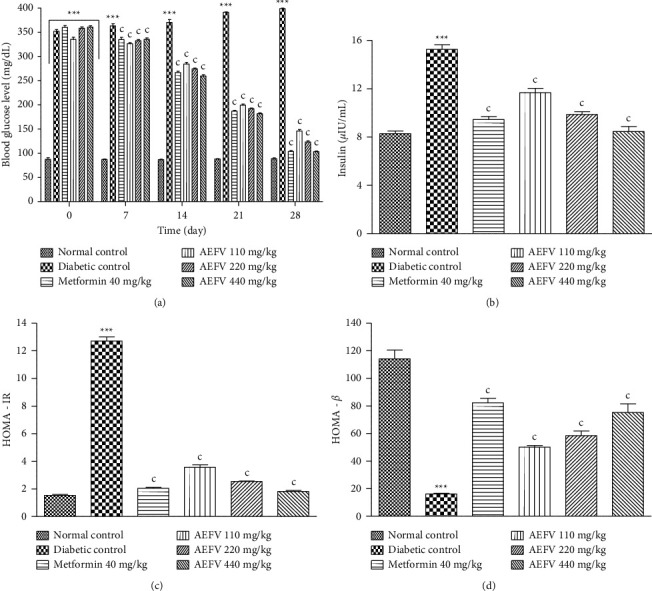
Effect of AEFV on the glycemia (a), insulin level (b), HOMA-IR (c), and HOMA-*ß* (d). Data analysis was performed by one-way ANOVA followed by Turkey's post hoc test. ^∗∗∗^*p* < 0.001 statistically significant compared with the normal control group. ^c^*p* < 0.001 statistically significant compared with the diabetic control group. AEFV: aqueous extract of the leaves of *Ficus vallis-choudae*; HOMA-IR: homeostasis model assessment of insulin resistance; HOMA-*β*: homeostasis model assessment of *β*-cell function.

Figure 1 shows the effect of AEFV on blood glucose (A), insulinemia (B), HOMA-IR (C), and HOMA-*β* (D). Compared with the normal control group, animals in the untreated diabetic group exhibited significantly elevated blood glucose levels (*p* < 0.001) throughout 4 weeks of treatment. However, in the groups of animals treated with AEFV and metformin, the blood glucose level decreased significantly (*p* < 0.001) from the 1st week until the end of the treatment, compared with the untreated diabetic control group.

Our results also showed a significant decrease (*p* < 0.001) in the HOMA-*ß* index and a significant increase (*p* < 0.001) in the serum insulin level and the HOMA-IR index, compared with the normal control group. On the other hand, treatment with metformin and at different doses of AEFV significantly reduced (*p* < 0.001) the HOMA-IR index and the serum insulin level and significantly increased (*p* < 0.001) the HOMA-*β* index of rats, compared with the untreated diabetic control (Figure 1).

### 3.4. Effect of AEFV on Lipid Profile and Cardiovascular Indices

The effects of AEFV on the lipid profile (TC, TG, LDL-c, VLDL-c, HDL-c) and the cardiovascular indices (AI, CRI, CI) are presented in Table 3. Compared with the normal control, we noted a significant increase (*p* < 0.001) in the levels of TC, TG, LDL-c, and VLDL-c and a significant decrease (*p* < 0.001) in the level of HDL-c in untreated diabetic animals. In contrast, the administration of metformin and AEFV resulted in a significant decrease (*p* < 0.001) in the levels of TC, TG, LDL-c, and VLDL-c and a significant increase (*p* < 0.001) in HDL-c levels, compared with the diabetic control group.

Furthermore, AI and CRA indices were significantly (*p* < 0.001) increased, while CI was significantly (*p* < 0.001) decreased in untreated diabetic animals, compared with animals in the normal control group. However, treatment with AEFV and metformin significantly reduced (*p* < 0.001) the AI and CRI indices and increased (*p* < 0.001) the CI index, compared with the untreated diabetic rats (Table 3).

### 3.5. Effect of AEFV on Serum AST, ALT, Urea, and Creatinine Levels

**Figure 2:**
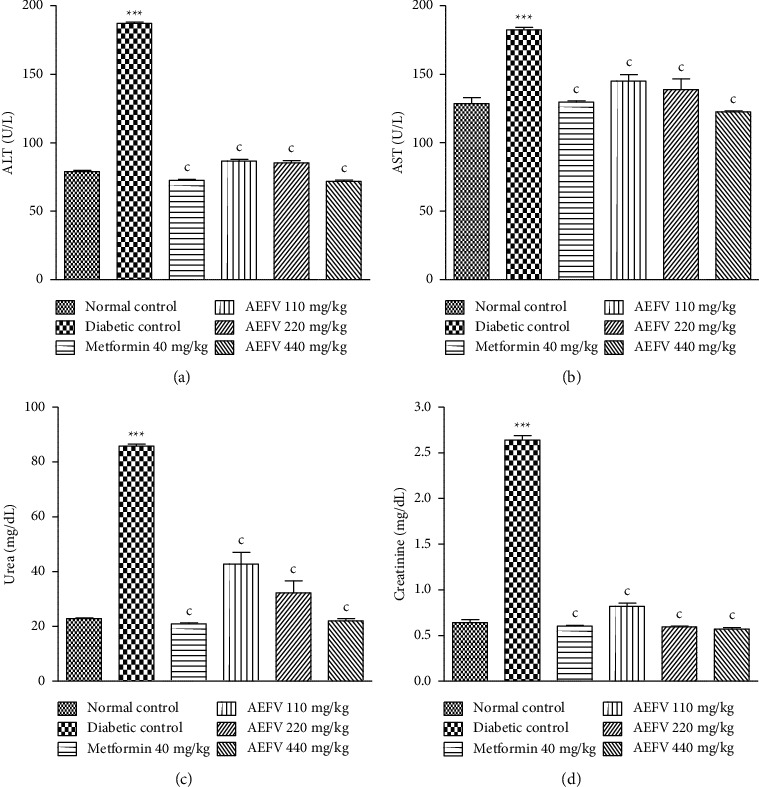
Effect of AEFV on the ALT (a), AST (b), urea (c), and creatinine (d). All results were expressed as mean ± standard derivation (*n* = 5). Data analysis was performed by one-way ANOVA followed by Turkey's post hoc test. ^∗∗∗^*p* < 0.001 statistically significant compared with the normal control group. ^c^*p* < 0.001 statistically significant compared with the diabetic control group. AEFV: aqueous extract of the leaves of *Ficus vallis-choudae*; ALT: alanine aminotransferase; AST: aspartate aminotransferase.

Figure 2 shows the effect of AEFV on markers of hepatic (AST and ALT) and renal (urea and creatinine) function. Compared with the normal control group, HFD and STZ resulted in an increase (*p* < 0.001) in the levels of AST, ALT, urea, and creatinine in the untreated diabetic rats. However, administration of AEFV and metformin to animals significantly (*p* < 0.001) attenuated the effects of HFD and STZ, compared with the untreated diabetic control group.

### 3.6. Effects of AEFV on Oxidative Stress Parameters

The effects of AEFV on the oxidative stress parameters (MDA, GSH, CAT, and SOD) are summarized in Table 4. Compared with the normal control group, the activity of SOD and CAT significantly (*p* < 0.001) decreased in the liver, kidneys, and heart of animals in the diabetic control group. However, metformin and the different doses of AEFV caused a significant (*p* < 0.001) increase in the activity of SOD and CAT in the liver, kidneys, and heart of the animals, compared with the diabetic control group.

Furthermore, there is a significant (*p* < 0.001) increase in the level of MDA and a significant (*p* < 0.05 to *p* < 0.001) decrease in the level of GSH in the liver, kidneys, and heart of the rats of the diabetic control group, compared with the normal control group. On the other hand, in the liver, kidneys, and heart of the animals that received metformin and the various doses of AEFV, there is a significant (*p* < 0.001) decrease in the MDA level and an increase (*p* < 0.05 to *p* < 0.001) in the GSH level, compared with the untreated diabetic control group.

## 4. Discussion

This work aimed to determine the phytochemical composition of AEFV and to evaluate its antidiabetic properties on a model of type 2 diabetes induced by HFD and a low dose of STZ. The results of phytochemical study reveal the presence of saponins, tannins, flavonoids, and total polyphenols in the AEFV. These results are in agreement with the work of Eddouks et al. [7] where a preliminary phytochemical study on the bark of *F. vallis-choudae* revealed the presence of flavonoids, glycosides, alkaloids, tannins, and saponins. The presence of different bioactive compounds could partly explain the pharmacological properties obtained in the present study.

The results of this study show that the body mass, BMI, and abdominal fat of animals fed HFD/STZ increased significantly. These results are in agreement with the work of Buettner et al. [35] who showed that diets based on lard cause hyperlipidemia and are the most obesogenic. In fact, in rodents fed a diet of lard, overfeeding is observed, which tends to decrease after 4 to 5 weeks and significantly increases the weight of these animals from the second week of the diet [35]. In addition, during a meal rich in fat, there is an increase in the storage of lipids in the form of triglycerides and therefore an increase in the size of adipose tissue. By increasing adiposity, the ability of the adipocyte to act as an endocrine cell will be affected and the secretion of several biologically active proteins such as leptin (a hormone that regulates energy balance) will then be impaired. This change causes a defect in leptin signaling, leading to excessive food intake and an increase in weight and adipose tissue and therefore BMI, which can lead to hyperlipidemia and later obesity [36]. This could be also explained by an increase in the blood level of free fatty acids and/or triglycerides resulting from a diet high in fat, thus leading to a decrease in insulin sensitivity. Lack of fatty acid oxidation leads to ectopic accumulation of triglycerides in the skeletal muscle and liver: this is called lipotoxicity [37].

Elevated plasma free fatty acid concentrations lead to dysfunctions in the insulin signaling cascade [38]. In addition, a study in rats fed a HFD suggests that ectopic accumulation of lipids is a better indicator of insulin resistance than the mass of adipose tissue [39]. Otherwise, STZ is a well-known cytotoxic chemical for pancreatic islet beta-cells and is extensively used to induce diabetes mellitus in animals. In the present study, intraperitoneal administration of STZ to the normal rats effectively induced diabetes.

STZ damage to the pancreatic islet of Langerhans *β*-cells leads to a low level of insulin production in diabetic rats, and it leads to the increase in the plasma glucose levels turning to diabetes. The drop in blood glucose after treatment with AEFV at doses of 110, 220, and 440 mg/kg and metformin could be explained by the improvement in insulin sensitivity on these sites of action, which would allow the use of glucose by peripheral tissues. The phytochemical tests carried out on the AEFV confirm the presence of bioactive compounds such as saponins, polyphenols, tannins, and flavonoids. These compounds can influence glucose metabolism by several mechanisms, such as inhibiting carbohydrate digestion and glucose uptake in the intestine, and stimulation of insulin secretion by pancreatic beta-cells, modulating the release of hepatic glucose, activation of the insulin receptor, and consumption of glucose in tissue insulin resistance and modulation of hepatic glucose utilization [40]. The mechanisms of the components of plant polyphenols against type 2 diabetes involve stimulation of cAMP, which increases exocytosis in *β*-cells, inhibition of insulin degradation processes, prevention of oxidative stress, regeneration of *β*-cells, cell repair and hypertrophy, and cell proliferation in the islets of Langerhans [41–43]. The insulin-secreting and insulin-sensitizing effects of AEFV observed in this study were further confirmed by the decrease in the HOMA-IR index and the decrease in the HOMA-*β* index, both of which are the main biomarkers of insulin.

Cholesterol is present in tissues and plasma lipoproteins. It exists as free cholesterol or as a cholesterol ester. It is synthesized from acetyl coenzyme-A and excreted from the body through the bile as cholesterol salts. HDL-c is often called “good cholesterol” because it is a lipoprotein that carries lipids from the periphery to the liver. Because of its relatively small size, compared with other lipoproteins, HDL can easily pass through the vascular endothelium and intima to bring back cholesterol accumulated in macrophages [44]. In addition, HDL has antioxidant properties that prevent the oxidation of LDL. LDL-c is a lipoprotein that transports cholesterol from the liver to peripheral tissues (extrahepatic) and is often referred to as “bad cholesterol.” Lipoproteins are responsible for transporting 65% to 70% of cholesterol [45].

High lipids in diabetic conditions increase the likelihood of cardiovascular disease. Our results on the lipid profile show an increase in the plasma concentration of TC, TG, VLDL-c, and LDL-c but a decrease in HDL-c concentration in the diabetic control group, compared with the rats of the normal control group. However, a decrease in TC, TG, LDL-c, AI, and CRI and an increase in HDL-c and CI were shown in the groups treated with AEFV (110, 220, and 440 mg/kg). The work of Miaffo et al. [46] also showed a decrease in the concentration of TC, TG, and LDL-c and a significant increase in the concentration of HDL-c following administration of the aqueous extract of the bark of *Vitellaria paradoxa* in rats subjected to HFD for 28 days. The hypocholesterolemic effect could be due to the ability of AEFV to inhibit cholesterol biosynthesis in the liver, decreased intestinal absorption of cholesterol, increased LDL receptors, and LDL uptake [47]. The triglycerides-lowering activity may be due to decreased fatty acid synthesis, increased LDL catabolism, increased tissue lipase activities, and acetyl-CoA carboxylase inhibition [47]. These results can be also explained by the presence of saponins in the AEFV confirmed by phytochemical tests, which have antihyperlipidemia, antihypercholesterolemia hypotensive, and cardiodepressive properties [48]. This antihypercholesterolemia effect of saponins may be due to the inhibition of acetyl-CoA cholesterol acyltransferase activity and the inhibitory effect of saponins on the absorption of cholesterol [49].

The liver plays a major role in the metabolism of carbohydrates and lipids. However, these liver functions are impaired in diabetes [50]. The main biomarkers used to determine cell and tissue damage in the liver are ALT and AST. One of the complications associated with diabetes is the increased concentration of urea and creatinine in the blood. The increase in the level of these parameters in the blood of diabetic rats indicates renal dysfunction [50]. The results obtained in this study show a significant increase in serum ALT and AST activity in untreated diabetic animals, which indicates liver damage. Likewise, the increase in the concentration of urea and creatinine in the same group of animals also indicates renal dysfunction. The AEFV decreased the serum activity of ALT and AST, which inhibited liver damage caused by HFD/STZ. Numerous studies have proven the effectiveness of certain herbs in improving kidney function [51]. The presence of flavonoids and saponins in our extract is responsible for improving liver and kidney function [52, 53]. The decrease in renal function biomarkers would be due to the decrease or inhibition of amino acid catabolism by AEFV.

Analysis of our results shows a significant increase in the tissue concentration of MDA in the liver, heart, and kidney, compared with the normal group. Furthermore, HFD/STZ significantly lowered the tissue concentration of GSH and SOD and CAT activity. Indeed, HFD/STZ induces an imbalance of oxidative status and then increases oxidative stress in rats. Yuzefovych et al. [54] have shown that a HFD leads to mitochondrial dysfunction correlated with increased oxidative stress in the kidneys, heart, and liver. In fact, numerous studies have shown that phenolic compounds from plant extracts constitute one of the main groups of compounds acting as primary antioxidants or free radical scavengers [55, 56]. Flavonoids are excellent scavengers of ROS and very good chelators of transition metals such as iron and copper. In addition, numerous studies have shown a positive correlation between the amounts of phenolic compounds and the antioxidant potential [57]. These chemical elements present in the AEFV are likely responsible for these effects [58].

## 5. Conclusion

The oral administration of AEFV in rats can improve chronic hyperglycemia and dyslipidemia and protect tissues of diabetic rats against damage induced by oxidative stress. These effects are thought to be due to the phytoconstituents present in the AEFV. This study provides evidence information to justify the use of *F. vallis-choudae* in the traditional treatment of type 2 diabetes and its complications. To complete this work, more in-depth and detailed studies will be performed later to isolate and identify the main active compounds and their mechanisms of action.

## Figures and Tables

**Table 1:** Content of bioactive components in the AEFV.

Bioactive compounds	Average
Polyphenols (mg gallic acid equivalent/g)	67.06 ± 0.11
Flavonoids (mg quercetin equivalent/g)	37.55 ± 0.11
Tannins (mg catechin equivalent/g)	31.80 ± 0.09
Saponins (mg diosgenin equivalent/g)	17.60 ± 0.05

All results were expressed as mean ± standard derivation (*n* = 3). Data analysis was performed by one-way ANOVA followed by Turkey's post hoc test.

**Table 2:** Effect of AEFV on body weight, BMI, and abdominal fat.

Parameters	Normal control	Diabetic control	Metformin		AEFV	
			(40 mg/kg)	110 mg/kg	220 mg/kg	440 mg/kg
Initial weight (g)	234.07 ± 2.70	378.78 ± 6.66^∗∗∗^	381.47 ± 4.12	370.37 ± 1.53	378.72 ± 5.12	365.25 ± 1.12
Final weight (g)	251.34 ± 2,85	374.62 ± 8.50^∗∗∗^	256.59 ± 2.11^c^	263.01 ± 3.45^c^	263.98 ± 4.32^c^	250.24 ± 2.77^c^
BMI (g/cm^2^)	0.66 ± 0.11	2.20 ± 0.08^∗∗∗^	0.68 ± 003^c^	0.75 ± 0.04^c^	0.78 ± 0.05^c^	0.66 ± 004^c^
Abdominal fat (g)	4.18 ± 0.09	9.27 ± 0.20^∗∗∗^	5.67 ± 0.24^c^	6.47 ± 0.02^c^	6.21 ± 0.06^c^	5.79 ± 0.18^c^

All results were expressed as mean ± standard derivation (*n* = 5). Data analysis was performed by one-way ANOVA followed by Turkey's post hoc test. ^∗∗∗^*p* < 0.001 statistically significant compared with the normal control group. ^c^*p* < 0.001 statistically significant compared with the diabetic control. BMI: body mass index; AEFV: aqueous extract of the leaves of *Ficus vallis-choudae*.

**Table 3:** Effect of AEFV on the lipids profile and cardiovascular indices.

Parameters	Normal control	Diabetic control	Metformin		AEFV	
			(40 mg/kg)	110 mg/kg	220 mg/kg	440 mg/kg
HDL-c (mg/dL)	47.16 ± 4.00	11.99 ± 0.54^∗∗∗^	42.61 ± 0.23^c^	39.88 ± 0.54^c^	40,29 ± 0.39^c^	41.75 ± 0.59^c^
LDL-c (mg/dL)	37.04 ± 0.52	94.47 ± 0.98^∗∗∗^	48.72 ± 0.98^c^	56.57 ± 2.98^c^	54,37 ± 2.84^c^	52.26 ± 0.44^c^
TG (mg/dL)	91.07 ± 3.97	158.53 ± 7.13^∗∗∗^	96.17 ± 0.49^c^	98.75 ± 7.14^c^	97,09 ± 6.78^c^	95.10 ± 4.52^c^
TC (mg/dL)	112.61 ± 2.63	202.20 ± 0.80^∗∗∗^	135.54 ± 4.68^c^	123.81 ± 2.80^c^	134,36 ± 2.16^c^	126.13 ± 5.67^c^
VLDL-c (mg/dL)	18.21 ± 0.79	31.70 ± 1.42^∗∗∗^	18.73 ± 0.57^c^	19.75 ± 1.42^c^	19,41 ± 1.35^c^	19.02 ± 0.90^c^
AI	1.44 ± 0.22	15.96 ± 0.80^∗∗∗^	2.18 ± 0.12^c^	2.10 ± 0.07^c^	2,34 ± 0.03^c^	2.01 ± 0.09^c^
CI	1.27 ± 0.10	0.12 ± 0.007^∗∗∗^	0.87 ± 0.02^c^	0.71 ± 0.04^c^	0,74 ± 0.05^c^	0.79 ± 0.01^c^
CRI	1.98 ± 0.21	13.35 ± 1.10^∗∗∗^	2.19 ± 0.07^c^	2.47 ± 0.17^c^	2,41 ± 0.16^c^	2.28 ± 0.12^c^

All results were expressed as mean ± standard derivation (*n* = 5). Data analysis was performed by one-way ANOVA followed by Turkey's post hoc test. ^∗∗∗^*p* < 0.001 statistically significant compared with the normal control group. ^c^*p* < 0.001 statistically significant compared with the diabetic control. AEFV: aqueous extract of the leaves of *Ficus vallis-choudae*; CI: cardioprotective index; TC: total cholesterol; TG: triglycerides; HDL-c: high-density lipoprotein cholesterol; LDL-c: low-density lipoprotein cholesterol; VLDL-c: very-low-density lipoprotein cholesterol; AI: atherogenic index; CRI: coronary artery risk index.

**Table 4:** Effect of AEFV on the parameters of oxidative stress.

Parameters	Organ	Normal control	Diabetic control	Metformin (40 mg/kg)	AEFV		
					110 mg/kg	220 mg/kg	440 mg/kg
SOD (U/mg)	Liver	95.23 ± 1.75	30.2 ± 1.83^∗∗∗^	93.83 ± 6.11^c^	91.78 ± 2.08^c^	97.19 ± 1.99^c^	106.14 ± 5.74^c^
	Heart	90.83 ± 2.70	46.91 ± 1.42^∗∗∗^	89.88 ± 3.13^c^	88.32 ± 1.55^c^	88.32 ± 2.51^c^	101.45 ± 3.23^c^
	Kidney	80.91 ± 2.70	61.21 ± 1.37^∗∗∗^	72.99 ± 1.40^a^	74.12 ± 4.30^a^	80.33 ± 1.45^c^	86.34 ± 2.41^c^

CAT (*µ*mol/mg)	Liver	57.40 ± 3.28	23.10 ± 1.27^∗∗∗^	59.57 ± 1.49^c^	55.27 ± 1.53^c^	60.97 ± 3.05^c^	72.17 ± 1.26^c^
	Heart	34.78 ± 2.28	15.75 ± 0.76^∗∗∗^	34.26 ± 1.07^c^	37.95 ± 0.76^c^	39.13 ± 1.00^c^	42.32 ± 1.36^c^
	Kidney	59.88 ± 1.91	29.71 ± 1.52^∗∗∗^	56.92 ± 1.20^c^	65.76 ± 2.10^c^	70.01 ± 4.51^c^	70.82 ± 0.65^c^

GSH (nmol/g)	Liver	6.73 ± 0.97	2.57 ± 0.37^∗∗∗^	5.31 ± 0.66^a^	3.76 ± 0.39	5.50 ± 0.36^a^	6.60 ± 0.34^b^
	Heart	6.30 ± 0.44	2.30 ± 0.37^∗∗∗^	5.41 ± 0.24^c^	4.35 ± 0.45^a^	5.63 ± 0.47^c^	6.30 ± 0.38^c^
	Kidney	5.49 ± 0.54	2.30 ± 0.36^∗∗∗^	4.45 ± 0.74	2.38 ± 0.32	4.49 ± 0.50	4.56 ± 0.35^a^

MDA (nmol/g)	Liver	31.48 ± 1.19	94.02 ± 1.38^∗∗∗^	24.19 ± 1.22^c^	34.01 ± 1.82^c^	43.53 ± 1.32^c^	21.39 ± 0.91^c^
	Heart	29.14 ± 1.38	58.74 ± 1.29^∗∗∗^	31.34 ± 1.68^c^	40.31 ± 2.25^c^	31.91 ± 2.16^c^	30.40 ± 1.29^c^
	Kidney	40.21 ± 1.35	69.87 ± 0.56^∗∗∗^	31.90 ± 0.92^c^	41.24 ± 1.75^c^	30.23 ± 1.57^c^	27.98 ± 0.88^c^

All results were expressed as mean ± standard derivation (*n* = 5). Data analysis was performed by one-way ANOVA followed by Turkey's post hoc test. ^∗∗∗^*p* < 0.001 statistically significant compared with the normal control group. ^a^p < 0.05, ^b^*p* < 0.01, ^c^*p* < 0.001 statistically significant compared with the untreated diabetic control. AEFV: aqueous extract of the leaves of *Ficus vallis-choudae*; SOD: superoxide dismutase; CAT: catalase; GSH: reduced glutathione; MDA: malondialdehyde.

## Data Availability

The data used to support the findings of this study are available from the corresponding author upon request.
